# *IFNL3* rs12980275 Polymorphism Predicts Septic Shock-Related Death in Patients Undergoing Major Surgery: A Retrospective Study

**DOI:** 10.3389/fmed.2020.00186

**Published:** 2020-05-14

**Authors:** Felipe Pérez-García, Maria Ángeles Jiménez-Sousa, Susana Soria, Pablo Jorge-Monjas, Amanda Fernández-Rodríguez, Esther Gómez-Sánchez, María Heredia-Rodríguez, Estefanía Gómez-Pesquera, Pedro Martínez-Paz, Eduardo Tamayo, Salvador Resino

**Affiliations:** ^1^Unidad de Infección Viral e Inmunidad, Centro Nacional de Microbiología, Instituto de Salud Carlos III, Majadahonda, Spain; ^2^Departamento de Anestesiología y Reanimación, Hospital Clínico Universitario, Valladolid, Spain; ^3^Departamento de Cirugía, Facultad de Medicina, Universidad de Valladolid, Valladolid, Spain

**Keywords:** IFNL3, rs12980275, SNP, sepsis, septic shock, survival, major surgery

## Abstract

Interferon lambda 3 (IFNL3, previously called IL-28B) is a cytokine with effects against viral and bacterial pathogens. We aimed to analyze the *IFNL3* rs12980275 SNP in patients who underwent major surgery, in order to establish its relationship with susceptibility to septic shock and septic shock-related death in these patients. We performed a case-control study on 376 patients to establish the association between *IFNL3* rs12980275 SNP and the susceptibility to develop septic shock. Besides, we performed a longitudinal study among 172 septic shock patients using survival analysis with one censoring point of 28-days mortality. The *IFNL3* rs12980275 polymorphism was genotyped by Agena Bioscience's MassARRAY platform. *IFNL3* rs12980275 polymorphism was not associated with higher susceptibility to infection and septic shock development. Regarding survival analysis, the Kaplan–Meier analysis showed that patients with *IFNL3* rs12980275 AA genotype had higher survival than patients with GG genotype (*p* = 0.003). The Cox regression analysis adjusted by the most relevant clinical and epidemiological characteristics showed that the GG genotype (recessive model) and the presence of the G allele (additive model) were associated with higher risk of death [adjusted hazard ratio (aHR) = 2.15, *p* = 0.034; aHR = 1.50, *p* = 0.030, respectively]. In conclusion, *IFNL3* rs12980275 polymorphism was associated with septic shock-related death in patients who underwent major surgery. The A allele was linked to protection, and the G allele was associated with an increased risk of death. This is a first preliminary study that suggests for the first time a role of *IFNL3* polymorphisms in the prognosis of septic shock.

## Introduction

Sepsis is a life-threatening disease, defined as a syndrome of organ dysfunction due to a dysregulated host response to an infection (1). Septic shock is defined as the most severe stage of sepsis, in which the underlying circulatory, cellular, and metabolic abnormalities are enough to increase mortality (1). In the last years, the incidence of sepsis has grown worldwide and has been reported as the most common cause of Intensive Care Unit (ICU) admission (2). This disease affects more frequently elderly patients, being especially common in patients with cancer or underlying immunosuppression (3). Regarding the incidence of sepsis, a recently published study estimated the incidence of sepsis about 48.9 million worldwide, estimating also sepsis-related death in 11 million (4). Although sepsis-related death is decreasing in the last years (4, 5), it remains unacceptably high, accounting as the most frequent cause of death in ICU patients, and constituting a significant cost for healthcare systems (6). An early and accurate diagnosis, in addition to adequate treatment, are crucial elements to reduce sepsis development (7). Moreover, the research of predictors of morbidity and mortality constitutes a priority in order to provide adequate management of patients with sepsis (8).

Sepsis is related to an inadequate immune response due to excessive inflammation that leads to organ dysfunction and multi-organ failure in the most severe cases (9). There are several pro-inflammatory cytokines (such as TNF, IL-1, IL-6, and IL-17), cell surface markers (for example, CD14) and transcription factors [nuclear factor-kappaB (NF-κB)] that are involved in the inflammatory response against infection and are also related to the pathophysiology of sepsis (9–11). The interferon lambda (IFNL or IFN-λ, also called type III IFN) family is composed of four IFNLs, IFNL1–4 (12). Interferon lambda 3 (IFNL3, previously called IL-28B) is a cytokine that modulates the immune response against viral and bacterial pathogens (12, 13). IFNL3 acts through its receptor (IFNLR1), which is mostly expressed by cells of epithelial origin and immune cells, inducing the expression of over 300 interferon-stimulated-genes (ISGs) (12, 13).

The vast majority of published research has focused on the contribution of IFNL3 to the anti-viral host defense, but IFNL3 has also been implicated in the anti-bacterial immune responses such as *Staphylococcus aureus, Pseudomonas aeruginosa*, and *Listeria monocytogenes* (13). Planet *et al*. (14) found that infection with the influenza virus and the subsequent activation of the IFNL3 signaling pathway led to an increased susceptibility to pulmonary infection by methicillin-resistant *Staphylococcus aureus*. Luo *et al*. (15) showed that IFNL3 levels were significantly higher in patients with sepsis and septic shock patients that died, compared with healthy controls and septic shock survivors, respectively. Besides, these authors showed in experimental septic models that the supplementation with IFNL3 worsened the outcome of sepsis, and the neutralization of IFNL3 activity with specific antibodies increased the sepsis survival and a decrease in neutrophil infiltration, (15). Additionally, Cohen and Prince (16) showed mice lacking IFNLR, when compared with controls, significantly improved the clearance of *S. aureus* and *P. aeruginosa*, and that it was also associated with a decrease in the duration of the inflammatory response.

The clinical impact of single nucleotide polymorphisms (SNPs) at the *IFNL3* gene has been described principally in the context of hepatitis C, being rs12979860, rs8099917, and rs12980275 the most-frequently related to spontaneous hepatitis C virus (HCV) clearance (17). These *IFNL3* SNPs are in high linkage disequilibrium (LD) between them and with other SNPs in *IFNL4* (12, 18). Moreover, *IFNL3* SNPs have also been associated with the prognosis of different viral infections (12), such as Andes virus, BK virus, cytomegalovirus, herpes simplex virus (HSV), and human T-lymphotropic leukemia virus (HTLV) type I, among others. However, the association of *IFNL3* SNPs with the dysregulated inflammatory response in patients with sepsis has not been reported so far.

Our study aimed to analyze the *IFNL3* rs12980275 SNP in patients who underwent major surgery in order to establish its relationship with susceptibility to septic shock and septic shock-related death.

## Materials and Methods

### Patients

We performed a case-control study, including patients from the Hospital Clínico Universitario of Valladolid (Spain). The study population consisted of patients that underwent major surgery, which was defined as any surgical procedure (abdominal or cardiac) that was performed under general anesthesia and respiratory assistance. A total of 376 patients were selected between April 2008 and November 2012 and stratified as follows: (a) 172 patients who developed an infection after the surgery (with positive culture) and a subsequent septic shock (SS-group); (b) 204 patients without infection but who developed a systemic inflammatory response syndrome (SIRS-group), which is a frequent condition after this kind of surgeries. Additionally, the survival at 28-day was analyzed in patients belonging to the SS-group.

The study was conducted according to the ethical requirements established by the Declaration of Helsinki. The Ethics Committee of Hospital Clínico Universitario de Valladolid and Instituto de Salud Carlos III approved the study. Written informed consent was provided by all participants before sample collection. When a patient was unable to sign, a family member or legal representative of the patient signed the consent.

### Clinical Data

Epidemiological and clinical data were retrieved from medical records. Emergency surgery was indicated for life-threatening conditions such as aortic dissection, heart and postoperative bleeding, and intestinal perforation.

Septic shock and SIRS diagnoses were established according to SCCM/ESICM/ACCP/ATS/SIS International Sepsis Definitions Conference criteria (19), which was in effect when the data and samples were collected. Subsequently, we updated them according to Sepsis-3 definitions (20). The septic shock diagnosis was made during the entire follow-up time post-surgery, and it was defined as an acute circulatory failure with persistent arterial hypotension that required vasopressor to maintain a mean arterial pressure of 65 mmHg or greater and serum lactate level greater than 18 mg/dL. SIRS diagnosis was made within the first 24 h post-surgery, as an inflammatory response to a noninfectious cause, and therefore, the infection in this group was ruled out. We selected the SIRS group as a control group with similar age and gender, which underwent to the same conditions as the case group (major surgery), but they did not develop sepsis. Finally, we confirmed that no patient had infection before major surgical intervention and all septic shock patients had a microbiologically confirmed infection.

Moreover, Acute Physiology and Chronic Health Evaluation (APACHE II score) and Sequential Organ Failure Assessment (SOFA score) were calculated for both groups, in order to assess the severity of the condition within the first 24 h following septic shock diagnosis. The choice of the most appropriate antibiotic therapy, as empiric treatment for sepsis, was based on our experience regarding the most common bacterial pathogens associated with sepsis in our medical ICU, and also according to international guidelines (21).

### DNA Genotyping

Blood samples for DNA genotyping were collected from all patients who underwent major surgery between 2008 and 2012. Total DNA from peripheral blood was extracted using the High Pure PCR Template Preparation kit (Roche Diagnostics GmbH, Mannheim, Germany). The rs12980275 SNP the was genotyped at the Spanish National Genotyping Center (CeGen; http://www.cegen.org/) by the Agena Bioscience's MassARRAY platform (San Diego, CA, USA) using the iPLEX® Gold assay design system (https://agenabio.com/products/massarray-dx/).

### Outcome Variables

We analyzed two main outcome variables: (i) susceptibility to develop septic shock (case-control study); (ii) mortality after diagnosis of septic shock (longitudinal sub-study) with one censoring point at 28-days, which is a commonly used primary endpoint for clinical trials that study the usefulness of new therapeutic approaches in severe sepsis patients (22).

### Statistical Analysis

Quantitative variables were expressed as the median and interquartile range (IQR), and categorical variables were expressed as absolute count (percentage). Comparisons between groups in the descriptive analysis of the study population were made using the Mann-Whitney *U*-test for continuous variables and the Chi-squared or two-tailed Fisher's exact test for categorical variables. A *p* ≤ 0.05 was considered significant. The *IFNL3* SNP was analyzed for deviation from the Hardy–Weinberg equilibrium (HWE), where *p* < 0.05 was considered to be statistically significant.

Regarding the genetic association study, analyses were carried out for dominant, recessive and additive models. In the case-control study (Septic shock-group vs. SIRS-group), logistic regression was performed to investigate the association between *IFNL3* SNP and the development of septic shock. In the longitudinal sub-study, a survival analysis was used to evaluate mortality at 28 days after septic shock diagnosis. Survival probabilities were estimated by the Kaplan–Meier product-limit method, and groups were compared using the log-rank test. The multivariate Cox regression test was adjusted by the most significant covariates, which were selected by a stepwise method (forward), from the following variables: age, gender, antibiotic treatment, peritonitis, hypertension, lactate, comorbidities [obesity, diabetes, chronic kidney disease, heart disease, chronic obstructive pulmonary disease (COPD), cancer, and liver disease], SOFA score, and type of surgery (emergency vs. scheduled; cardiac vs. abdominal). Statistical analysis was performed using Stata/IC 13.1 (StataCorp, Texas, USA).

## Results

### Susceptibility to Septic Shock

The demographic and clinical characteristics of the selected 376 patients are summarized in Table 1. Briefly, patients of the SS-group (*n* = 172) had higher percentages of chronic kidney disease, higher values of SOFA and APACHE II score, and required abdominal and emergency surgery more frequently. On the other hand, patients of the SIRS-group (*n* = 204) presented higher percentages of heart disease, cancer, and cardiac surgery.

**Table 1:** Demographic and clinical characteristics of patients with SIRS (SIRS-group) and with septic shock (SS group) who underwent major surgery.

**Characteristics**	**SIRS-group**	**SS-group**	***p*-value**
No. of patients	204	172	–
Gender (male)	130 (63.7%)	110 (64.0%)	1.000
Age (years)	72 (65–78)	74 (63–80)	0.526
**Underlying conditions**			
Smoker	26 (12.8%)	30 (17.4%)	0.245
Alcoholism	6 (2.9%)	10 (5.8%)	0.204
Obesity	22 (10.8%)	26 (15.1%)	0.219
Diabetes	40 (19.6%)	22 (12.8%)	0.094
Heart disease	118 (57.8%)	79 (45.9%)	**0.023**
COPD	29 (14.2%)	28 (16.3%)	0.665
Hypertension	123 (60.3%)	96 (55.8%)	0.402
Chronic kidney disease	12 (5.9%)	27 (15.7%)	**0.002**
Cancer	76 (37.3%)	41 (23.8%)	**0.005**
Liver disease	3 (1.5%)	7 (4.1%)	0.196
**Type of surgery**			
Cardiac (vs. abdominal)	112 (54.9%)	71 (41.3%)	**0.010**
Emergency (vs. scheduled)	19 (9.3%)	107 (62.2%)	**<0.001**
**Severity indexes**			
SOFA score	3 (3, 4)	9 (7–11)	**<0.001**
APACHE II score	9 (8–10)	17 (13–20)	**<0.001**

Statistics: Values are expressed as median (percentile 25-percentile 75) and absolute count (percentage). *P-values were calculated by Fisher's exact test for categorical variables and Mann-Whitney test for continuous variables. Significant differences are shown in bold. Abbreviations: SIRS, systemic inflammatory response syndrome; p-value, level of significance; COPD, Chronic obstructive pulmonary disease; SOFA, sequential organ failure assessment; APACHE, acute physiology and chronic health evaluation.

**Figure 1:**
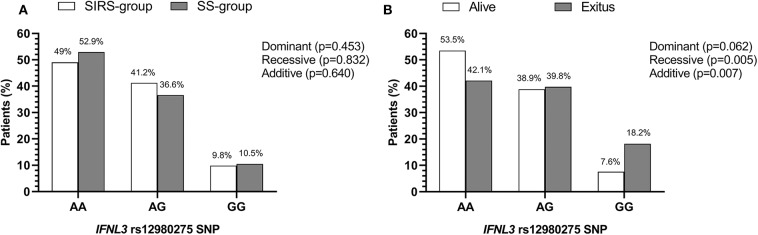
Genetic association of *IFNL3* rs12980275 polymorphism with susceptibility to septic shock and shock-related death in patients who underwent major cardiac or abdominal surgery. **(A)** SIRS-group vs. SS-group. **(B)** Alive vs. exitus patients. Statistics: *P*-value was calculated by logistic regression analysis. Abbreviations: IFNL3, Interferon lambda 3; SIRS, systemic inflammatory response syndrome; SS, septic shock; SNP, single nucleotide polymorphism.

The rs12980275 polymorphism had a minor allelic frequency (G vs. A) higher than 10% and fulfilled the HWE (*p* > 0.05). The genotypic frequencies of *IFNL3* rs12980275 polymorphism were very similar between the SS-group and the SIRS-group (Figure 1A).

The rs12980275 polymorphism was not associated with susceptibility to the development of septic shock, regardless of the inheritance model analyzed. For this reason, it was omitted from the multivariate logistic regression analysis.

### Risk of Death in Patients With Septic Shock

The baseline characteristics of septic shock patients stratified by alive vs. exitus are shown in Supplementary Table 1. Patients who died were older, had higher lactate values and SOFA and APACHE II scores, and higher percentages of emergency surgery and chronic kidney disease (*p* < 0.05). Ninety-seven percent of patients had adequate initial empirical treatment according to the antibiogram data. Moreover, all patients survived in the SIRS group during the follow-up period (28 days).

**Figure 2:**
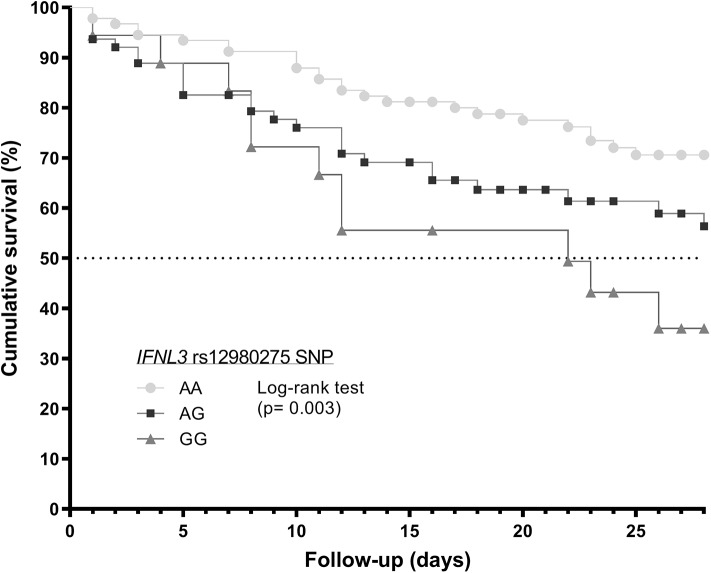
Survival analysis (Kaplan–Meier curve) regarding to *IFNL3* rs12980275 polymorphism in septic shock patients who underwent major cardiac or abdominal surgery. Statistics: *P*-value was calculated by log-rank test. Abbreviations: IFNL3, Interferon lambda 3; SNP, single nucleotide polymorphism.

The genotypic frequency of *IFNL3* rs12980275 polymorphism was different between alive and dead patients with septic shock during the first 28 days after septic shock diagnosis (Figure 1B), according to the three inheritance models analyzed, which suggests an association of this polymorphism with death at 28 days in ICU. The Kaplan-Meier analysis (Figure 2 and Table 2) showed that patients with *IFNL3* rs12980275 AA genotype had higher survival than patients with GG genotype (*p* = 0.003). The Cox regression analysis adjusted by the most relevant clinical and epidemiological characteristics showed that the G allele [either in a recessive model (GG) or additive (G)] was associated with a higher risk of death [adjusted hazard ratio (aHR) = 2.15, *p* = 0.034; aHR = 1.50, *p* = 0.030, respectively; Table 2].

**Table 2:** Survival probabilities at 28 days (Kaplan–Meier product-limit method) and risk of death in septic shock patients (Cox regression) who underwent major cardiac or abdominal surgery according to *IL-28B* rs12980275 SNP.

		**Kaplan–Meier**			**Cox**	
**Model**	**Genotype**	***N***	**Deaths**	***p*-value**	**aHR (95%CI)**	***p*-value**
Dominant	AA	91	25 (27.5%)	**0.009**	1.54 (0.91–2.61)	0.107
	AG/GG	81	36 (44.4%)			
Recessive	AA/AG	154	50 (32.5%)	**0.019**	2.15 (1.06–4.35)	**0.034**
	GG	18	11 (61.1%)			
Additive	AA	91	25 (27.5%)	**0.003**	1.50 (1.04–2.18)	**0.030**
	AG	63	25 (39.7%)			
	GG	18	11 (61.1%)			

*Statistics: values are expressed as absolute count and percentage, and hazard ratio and 95% confidence interval. Significant differences are shown in bold. Abbreviations: aHR, adjusted hazard ratio; 95%CI, 95% confidence interval; p-value, level of significance; SNP, single nucleotide polymorphism; Cox, regression analysis adjusted by the most significant clinical and epidemiological characteristics*.

## Discussion

Our study shows that *IFNL3* rs12980275 polymorphism was not associated with susceptibility to the development of septic shock, but a significant association with the risk of death at 28 days in septic shock patients was found. The A allele was linked to protection and the G allele was associated with an increased risk of death. To the best of our knowledge, this is the first study that points out the relationship between *IFNL3* SNPs and death in septic shock patients.

*IFNL* SNPs at transcription factor binding sites, promoter regions, methylation sites, and frameshift mutations, seem to modulate IFNL expression (12, 18). Specifically, rs12980275 is located in a downstream region of *IFNL3* and *IFNL4* genes and could be implicated in the regulation of gene expression since rs12980275 seems to influence the chromatin state (23). However, the search for the causal *IFNL* mutation is complicated, as SNPs within *IFNL3* gene are in high LD with other SNPs at *IFNL4*. Thus, the relationship between *IFNL3* SNPs and plasma IFNL3 levels is still not clear, since the different authors that have addressed this issue have reached discrepant results (12, 18). Moreover, minor alleles at *IFNL3* SNPs have been associated with ISG expression, suggesting that the immune response depends on the *IFNL3* SNPs (12, 13, 18). This last finding seems to be related to other SNPs that regulate the production of IFNL4 (18). As proposed by Bhushan and Chinnaswamy (18), the presence of the major *IFNL3* alleles (rs12979860 C allele and others in LD such as rs12980275 A allele) are linked to the *IFNL4* rs11322783 (previously called rs368234815) TT allele, which avoids the expression of a functional IFNL4 protein, generating a set of ISGs that could result in a protective phenotype against infection (18). On the other hand, the presence of the minor *IFNL3* alleles (rs12979860 T allele and others in LD such as rs12980275 G allele) are linked to the *IFNL4* rs11322783 ΔG allele, which is responsible of a fully functional IFNL4 protein expression, generating another set of ISGs that could result in a risk phenotype against infection (18). Therefore, taking into account what was discussed above, we can hypothesize that the *IFNL3* rs12980275 AA genotype may be associated with a better outcome in patients with septic shock, decreasing the risk of death at 28 days.

Sepsis is a complex syndrome related to an excessive and inadequate immune response that may lead to organ dysfunction and multi-organ failure (10). Therefore, IFNL could be one of the mechanisms that modulate the inflammation and control pathogens in septic patients. For major surgery patients, the study of their genetic profile and the implementation of predictive models could help clinicians to identify those patients with septic shock related death. However, this hypothesis must be confirmed by further studies covering the analysis of serum levels of cytokines, gene expression products, and *IFNL3* polymorphisms in patients with sepsis.

## Limitations of the Study

Firstly, this study had a retrospective design and it was conducted in one single hospital, only including patients that underwent major cardiac or abdominal surgery. Due to this fact, our conclusions cannot be generalized to other septic patients. In order to overcome these limitations, further multi-centric studies, including patients from all kinds of ICUs, would be necessary. Secondly, we have a low sample size which limited the statistical power of this analysis and could explain the lack of association of *IFNL3* rs12980275 SNP with susceptibility to septic shock. Additionally, the low sample size could also increase the rate of false positives. Thirdly, regarding the survival analysis, it was performed with only one censoring point of 28-days mortality, but this censoring point day 28 is more appropriate than others (such as 7 or 90 days) to establish sepsis-related death (24). Fourthly, as a high LD exists between *IFNL1-4* polymorphisms, we interpreted that our results could be extrapolated to the linked *IFNL* SNPs. However, additional studies analyzing other SNPs in *IFNL* would be needed to confirm our results. Finally, another interesting analysis that we have not performed was to corroborate our results, that is, to identify the biological function of the individual SNPs of IFNL3/4. However, these research are out of the scope of our study and also constitute tasks that are quite complex and that have been investigated for a long time, more than a decade, by a large number of scientists without reaching conclusive results (12, 13, 18).

## Conclusions

In conclusion, *IFNL3* rs12980275 polymorphism was associated with septic shock-related death in patients who underwent major surgery. This is a first preliminary study that suggests for the first time a role of *IFNL3* polymorphisms in the prognosis of septic shock. However, since the statistical power of this study was low, more research is needed to corroborate our findings.

## Data Availability Statement

The raw data supporting the conclusions of this article will be made available by the authors, without undue reservation, to any qualified researcher.

## Ethics Statement

The study was conducted according to the ethical requirements established by the Declaration of Helsinki. The Ethics Committee of Hospital Clínico Universitario (Valladolid) and Instituto de Salud Carlos III (Majadahonda) approved the study. Written informed consent was provided by all participants before sample collection. When a patient was unable to sign, a family member or legal representative of the patient signed the consent.

## Author Contributions

ET and SR: funding body. MJ-S, ET, and SR: study concept and design. SS, PJ-M, EG-S, MH-R, EG-P, PM-P, and ET: patients' selection and clinical data acquisition. MJ-S: sample preparation, DNA isolation and genotyping. FP-G, MJ-S, and SR: statistical analysis and interpretation of data and writing of the manuscript. AF-R and ET: critical revision of the manuscript for relevant intellectual content. SR: supervision and visualization. All authors read and approved the final manuscript.

## Conflict of Interest

The authors declare that the research was conducted in the absence of any commercial or financial relationships that could be construed as a potential conflict of interest.
