# Supplementary material for: IFNL3 rs12980275 Polymorphism Predicts Septic Shock-Related Death in Patients Undergoing Major Surgery: A Retrospective Study
Source: Front Med (Lausanne). 2020 May 14;7:186. doi: 10.3389/fmed.2020.00186 (PMC7239994; doi:10.3389/fmed.2020.00186)
Supplement: Supplementary file 1 [file Table_1.doc]

**Supplementary Table 1.** Demographic and clinical characteristics of septic shock patients who underwent major surgery stratified by alive versus exitus.

| **Characteristics** | **Alive** | **Exitus** | ***p*-value** |
| --- | --- | --- | --- |
| No. patients | 111 | 61 | - |
| Gender (male) | 76 (68.5%) | 34 (55.7%) | 0.101 |
| Age (years) | 72 (61-79) | 78 (69-81) | **0.015** |
| **Underlying conditions** |  |  |  |
| Smoker | 20 (18.0%) | 10 (16.4%) | 0.837 |
| Alcoholism | 5 (4.5%) | 5 (8.2%) | 0.329 |
| Obesity | 14 (12.6%) | 12 (19.7%) | 0.267 |
| Diabetes | 17 (15.3%) | 5 (8.2%) | 0.235 |
| Heart disease | 48 (43.2%) | 31 (50.8%) | 0.424 |
| COPD | 18 (16.2%) | 10 (16.4%) | 1.000 |
| Hypertension | 60 (54.1%) | 36 (59.0%) | 0.630 |
| Chronic kidney disease | 11 (9.9%) | 16 (26.2%) | **0.008** |
| Cancer | 25 (22.5%) | 16 (26.2%) | 0.581 |
| Liver disease | 3 (2.7%) | 4 (6.6%) | 0.247 |
| **Surgery** |  |  |  |
| Cardiac (versus abdominal) | 55 (49.5%) | 16 (26.2%) | **0.004** |
| Emergency (versus scheduled) | 58 (52.3%) | 49 (80.3%) | **<0.001** |
| **Severity** |  |  |  |
| Time to septic shock (days) | 2 (0-6) | 0 (0-2) | **<0.001** |
| Late septic shock (> 4 days) | 31 (27.9%) | 7 (11.5%) | **0.013** |
| White Blood Cell (*10^3^ cells/mm^3^) | 14.6  (9.6-20.1) | 15.0  (9.2-24.5) | 0.749 |
| C-Reactive protein (mg/L) | 238.0  (122.0-311.0) | 236.5  (166.1-285.9) | 0.908 |
| Procalcitonin (ng/mL) | 4.9 (1.1-18.0) | 5.0 (2.0-22.0) | 0.393 |
| SOFA score | 9 (7-10) | 9 (7-11) | 0.218 |
| APACHE II score | 15 (13-19) | 18 (15-22) | **0.001** |
| **Microorganism isolated** |  |  |  |
| Gram-positive | 60 (54.1%) | 24 (39.3%) | 0.080 |
| Gram-negative | 65 (58.6%) | 28 (45.9%) | 0.150 |
| Fungus | 20 (18.0%) | 16 (26.2%) | 0.241 |
| **Site of infection** |  |  |  |
| Catheter-related bacteremia | 49 (44.1%) | 11 (18.0%) | **<0.001** |
| Surgical site infection | 13 (11.7%) | 6 (9.8%) | 0.720 |
| Urinary tract infection | 8 (10.8%) | 11 (11.2%) | 0.803 |
| Endocarditis | 4 (3.6%) | 6 (9.8%) | 0.169 |
| Peritonitis | 43 (38.7%) | 38 (62.3%) | **0.004** |
| Pneumonia | 62 (55.9%) | 21 (34.4%) | **0.010** |
| Adequate initial empirical treatment | 108 (97.3%) | 59 (96.7%) | 1.000 |

**Statistics:** Values are expressed as median (percentile 25-percentile 75) and absolute count (percentage). (*), *p*-values were calculated by Fisher's exact test for categorical variables and Mann-Whitney test for continuous variables. Significant differences are shown in bold. Note that patients may have had more than one organism cultured. **Abbreviations**: p-value: level of significance; COPD: Chronic obstructive pulmonary disease; SOFA: sequential organ failure assessment; APACHE: acute physiology and chronic health evaluation.
